# Epileptic MEG Spike Detection Using Statistical Features and Genetic Programming with KNN

**DOI:** 10.1155/2017/3035606

**Published:** 2017-10-01

**Authors:** Turky N. Alotaiby, Saud R. Alrshoud, Saleh A. Alshebeili, Majed H. Alhumaid, Waleed M. Alsabhan

**Affiliations:** ^1^KACST, Riyadh, Saudi Arabia; ^2^KACST-TIC in Radio Frequency and Photonics for the e-Society (RFTONICS), Electrical Engineering Department, King Saud University, Riyadh, Saudi Arabia; ^3^King Fahad Medical City, Riyadh, Saudi Arabia

## Abstract

Epilepsy is a neurological disorder that affects millions of people worldwide. Monitoring the brain activities and identifying the seizure source which starts with spike detection are important steps for epilepsy treatment. Magnetoencephalography (MEG) is an emerging epileptic diagnostic tool with high-density sensors; this makes manual analysis a challenging task due to the vast amount of MEG data. This paper explores the use of eight statistical features and genetic programing (GP) with the K-nearest neighbor (KNN) for interictal spike detection. The proposed method is comprised of three stages: preprocessing, genetic programming-based feature generation, and classification. The effectiveness of the proposed approach has been evaluated using real MEG data obtained from 28 epileptic patients. It has achieved a 91.75% average sensitivity and 92.99% average specificity.

## 1. Introduction

**Figure 1:**
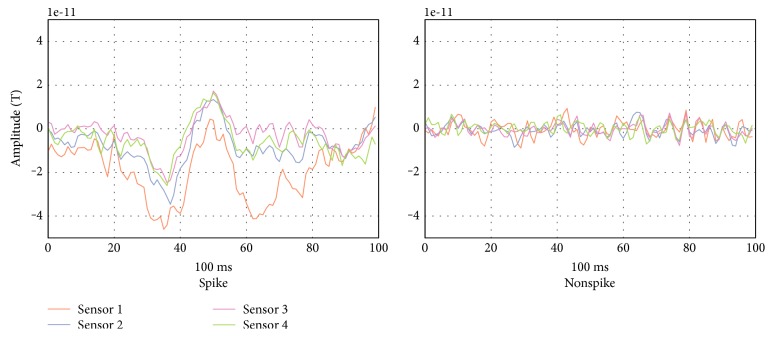
MEG interictal spiky and nonspiky segments.

Epilepsy is a neurological brain disorder characterized by recurrent seizures. It has been estimated to affect around 1% of the world's population [1]. Yearly, some 200,000 new cases of epilepsy are being diagnosed in the United States and about 2.4 million new cases globally [2]. Patients with epilepsy can harm themselves and/or others during a seizure episode. Different technologies have been used for monitoring and analyzing brain activities, such as EEG and FMRI. The former is considered the primary tool for epilepsy diagnosis. Due to the recent advancement in technology, magnetoencephalography (MEG), a neuroimaging technique that records the magnetic field generated by the brain's electrical current using superconducting quantum interference devices (SQUIDs), has become a new information source for brain activities, and it is used clinically in different applications [3, 4] such as a diagnostic tool in pre- and postsurgical assessments [5–7]. Surgical intervention is preceded by seizure localization, which starts with spike detection. Researchers have developed different methods for interictal EEG spike detection [8–16] and criteria for defining EEG spikes [10]. However, until now, there has been no formal definition of MEG spikes [17]. Direct application of EEG spike criteria on MEG spikes may not be always valid [18]. Ossenblok et al. [19] found that MEG spikes are more distinguishable from the background activities and sharper. One explanation of the differences between EEG and MEG spikes may be attributable to the fact that a MEG signal is not distorted or attenuated by the materials' conductivity that lie between the brain and the recording device [20–26]. In fact, EEG and MEG data are complementary and should not be considered as mutually exclusive. Most commonly, in clinical practice, interictal epileptic spikes are manually identified and marked by the neurologist through visual inspection of the MEG (about 300 sensors) recording, which is a very tedious, time-consuming, and subjective method [8, 27–29]. A MEG reading session can take hours due to the vast amount of MEG data. Thus, an objective, reliable, and automatic method for the detection of interictal spikes is strongly desired in clinical practices. In this work, we develop a novel MEG epileptic spike detection algorithm by segmenting the data into overlapping segments and extract eight easy to calculate statistical features: the maximum and minimum values, mean, standard deviation, median, interquartile range, kurtosis, and skewness. Then, the genetic programming (GP) is used to improve the discrimination performance of the K-nearest neighbor (KNN) classifier.  Figure 1 represents a spiky and nonspiky MEG segments taken from an epileptic patient.

MEG data drawn from 28 epileptic patients is used to evaluate the performance of the proposed method. The obtained results show that it has better performance compared with the previously published work [30, 31].

This paper is organized as follows. After the introduction in Section 1, Section 2 presents an overview of GP. The clinical MEG data used to evaluate the proposed methodology is described in Section 3. The spike detection methodology is presented in Section 4.  Section 5 presents and compares our results with those of other MEG spike detection algorithms. Finally, the conclusions are drawn in Section 6.

## 2. Genetic Programming

**Figure 2:**
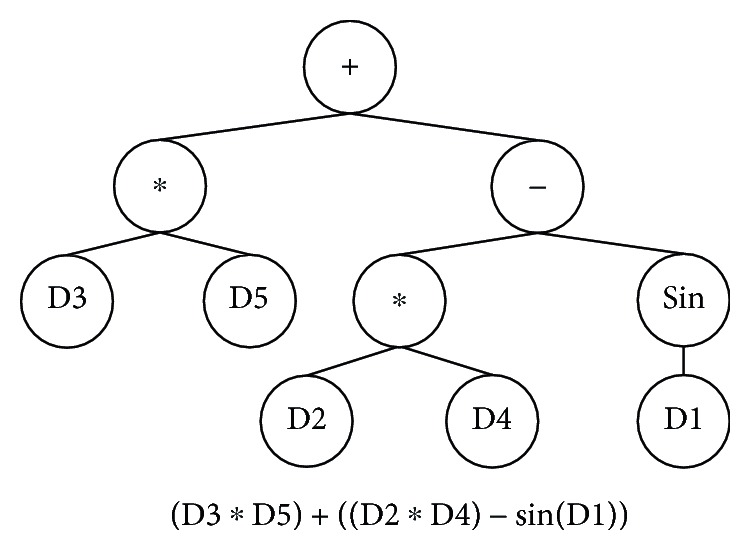
Example of tree structure formula.

GP is a well-known evolutionary algorithm used to optimize solutions using biological evolution mechanisms [32], and it has been used for the classification of data and signals [33, 34]. GP is a population-based algorithm, which means that it starts with multiple initial solutions and tries to generate new and better solutions. Each solution (i.e., each individual in the population) is a mathematical formula that is represented as a tree. In the tree-based GP, the tree's terminal nodes are the input data and the internal nodes are functions. A binary function node has two “children,” while a unary operator node has one “child.” The output node gives the output of the tree.  Figure 2 shows an example of the mathematical tree structure formula, where D1 to D5 are five different inputs of a problem.

Each tree structure formula generated by the GP is evaluated using the fitness function, which is defined based on the problem, to give a value that represents the significance of each tree formula for solving the given problem. This value is called the “fitness value.” The fitness function design is problem-dependent. When the formula with the higher fitness value is required, then it is known as a maximization problem. Otherwise, it is a minimization problem. In each generation of the GP, it derives new trees from the best trees found by implementing some GP operations. Crossover and mutation are the most commonly used GP operations to generate new trees. The crossover is performed by swapping randomly selected branches of a tree with other randomly selected branches from other trees that are from the current generation. This operation generates two new offsprings. The mutation is performed by replacing a randomly selected branch of a tree with a new randomly generated tree.

The following steps describe the common procedure of a GP:

1. Initialization: It generates an initial population that represents the first generation. This is usually done with the creation of random solutions.
2. GP operations (crossover and mutation): It operates on the population trees depending on a probability (crossover probability and mutation probability).
3. Evaluation: It calculates the fitness value of the new individuals using a fitness function. The main objective of the function is to give a value to each individual, which represents how good it is. This value is then used to identify the best individual.
4. Selection: It transfers trees with the best fitness values to the next generation. The trees are selected from both the current and previous generations.
5. Termination: If the termination condition has been met, the algorithm stops and reports the fittest tree (or best solution) that it has found. The termination condition could be a certain number of generations or a certain fitness value. If the termination condition has not been met, the algorithm creates a new generation by repeating steps 2 to 5.

## 3. Clinical MEG Data

The proposed methodology tested on high-quality MEG data recorded at the National Neuro Institute (NNI), the King Fahad Medical City (KFMC) in Riyadh, Saudi Arabia uses Elekta Neuromag in a shielded room, in which there are 306 sensors (102 magnetometer and 204 gradiometer). These sensors are categorized and distributed over the head according to the eight brain regions (left temporal, right temporal, left frontal, right frontal, left parietal, right parietal, left occipital, and right occipital). Each region has 26 channels, except for the left occipital and right occipital regions, which have 24 channels only. In our development, data of the 204 gradiometer sensors have been used.

Magnetic brain activity of 28 epileptic patients (patients' ages range between 14 and 43 years) in resting-state supine position at a sampling frequency of 1000 Hz and band-pass of 0.03 to 330 Hz is recorded in separate sessions of 15 minutes each. In synchrony with the MEG, 21-channel electroencephalogram (EEG, international 10–20 system), electrooculogram (EOG), and electrocardiogram (ECG) were also recorded. The MEG signals were then band-pass filtered between 1 and 50 Hz for visual inspection and examined together with the concurrent 21-channel EEG data by at least one MEG/EEG technician and one neurology consultant, following the standard principles established for clinical EEG. There are no more than three sessions for each patient, with 433 spikes in total. The experts determine the brain region and spikes' duration (start–end) by visual inspection. The dataset contains two patients with right frontal, one patient with left frontal, two patients with right parietal, three patients with left parietal, 11 patients with right temporal, and nine patients with left temporal, with 26/24 channels of each.

## 4. Spike Detection Methodology

**Figure 3:**
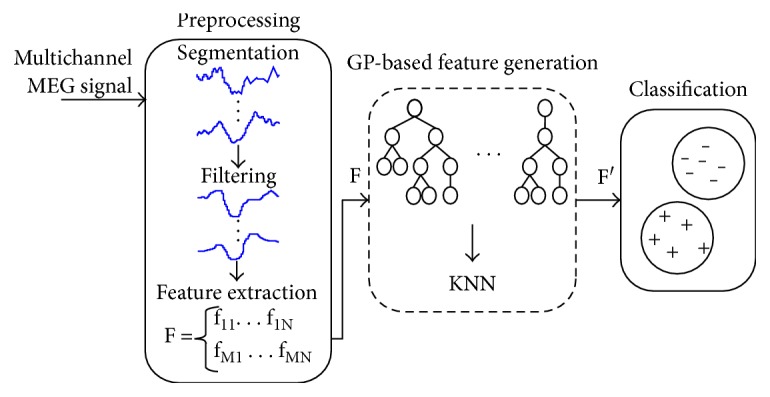
Spike detection methodology overview.

The proposed spike detection methodology comprises three stages: preprocessing, GP-based feature generation, and classification.  Figure 3 depicts an overview of the methodology. The next subsections detail each stage.

### 4.1. Preprocessing Stage

**Figure 4:**
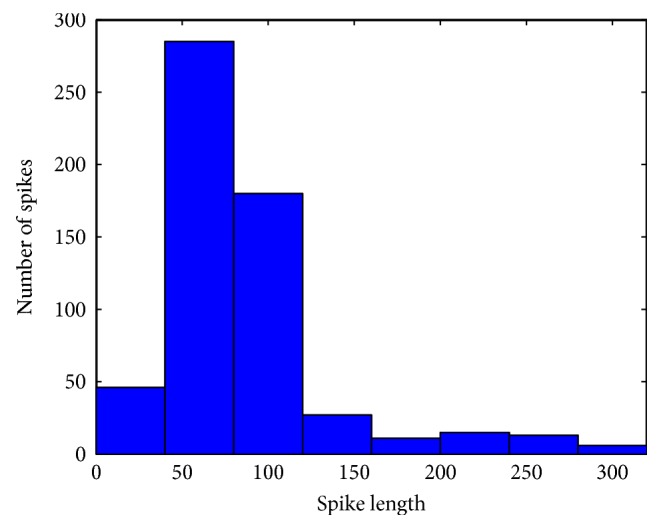
Spikes' duration distribution.

In this stage, three tasks are performed: data segmentation, data smoothing, and feature extraction. The multichannel MEG signal is segmented into epochs of *N* = 100 ms with 50% overlapping between two successive epochs. *N* is selected based on the analysis of spike duration in all the epileptic patients used in this study.  Figure 4 shows the histogram of the spikes' durations. A median filter is then applied on each segment to smoothen the signal. From each segment, eight statistical features are extracted: the maximum and minimum values, mean, standard deviation, median, interquartile range, kurtosis, and skewness.

The mean and median are the most used measures for central tendency. Equation (1) is used to obtain the mean (*μ*), where *X*_*i*_ is the *i*th data point of the segment and *N* is the length of the segment. To measure the statistical dispersion of segment data points, we used the standard deviation and the interquartile range. Equation (2) is used to calculate the standard deviation (*σ*). The interquartile range (IQR) is the difference between the median of the lower-half data points (Q1) and the median of the upper-half data points (Q3). Kurtosis and skewness are measurements of the shape of the distribution of data. Kurtosis measures the curviness of the data distribution and indicates whether it is peaked or flat. Skewness measures the symmetry of the data distribution around the mean. Equations (3) and (4) are performed to calculate the kurtosis and skewness, respectively [35].

(1) $$\begin{array}{c} \mu =\frac{1}{N}\overset{N}{\underset{i=1}{\sum }}X_{i}. \end{array}$$

(2) $$\begin{array}{c} \sigma =\sqrt{\frac{1}{N-1}\overset{N}{\underset{i=1}{\sum }}{\left(X_{i}-\mu \right)}^{2}}. \end{array}$$

(3) $$\begin{array}{c} kurtosis=\frac{1}{\left(N-1\right)\sigma^{4}}\overset{N}{\underset{i=1}{\sum }}{\left(X_{i}-\mu \right)}^{4}. \end{array}$$

(4) $$\begin{array}{c} skewness=\frac{1}{\left(N-1\right)\sigma^{3}}\overset{N}{\underset{i=1}{\sum }}{\left(X_{i}-\mu \right)}^{3}. \end{array}$$

### 4.2. GP-Based Feature Generation

In this stage, the GP algorithm is applied to automatically create the new feature (best mathematical formula) that combines selected features. Different input feature (max, min, mean, standard deviation, median, IQR, kurtosis, and skewness) combinations are tried by GP in the form of individuals.  Table 1 presents the GP's input values that returned the best results. The six mathematical operations used are summation, subtraction, multiplication, sine, cosine, and natural logarithm. The GP starts with 25 random individuals (first generation). Its operations (crossover and mutation) are applied on the individuals, according to a probability. The GP operations' probabilities change, depending on the results of the previous generation. The initial adaptive probabilities for crossover and mutation are 90% and 10%, respectively. The new generated trees (formulas) are limited to 25 levels. Therefore, the crossover and the mutation are not allowed to break this limit. The fittest 25 individuals (of the parents and the children) are selected as the new generation. In other words, the 25 formulas that give the best fitness values are selected. The GP algorithm terminates after 100 generations, and the best individual (formula) is reported.

The fitness function used to evaluate the formulas is the K-nearest neighbor (KNN) [36], which is a supervised learning model. It uses preclassified records to classify unknown records based on the extracted features. KNN classifies a new record based on the majority of the “k”-similar preclassified records. Usually, the similarity measurement is a distance function (e.g., Euclidean function). We used a small set of data in order to use KNN as a fitness function. This data was divided into two groups. The first group of segments was used as reference points for the KNN, while the second group was used to validate the formulas. Each group has an equal number of spike and nonspike segments. The fitness value is the error rate of the KNN classification results. The GP formula with the minimum error rate (best formula) is selected to be used in the next stage. GPLab toolbox has been used for the experiments in this work [37].

### 4.3. Classification

After finding the best mathematical formula (with the lowest error rate), KNN algorithm is then used to classify data of each channel of a particular region of the brain using the same reference points mentioned in stage two. Note that the similarity measure used by the KNN approach is based on the calculated features. In particular, the KNN algorithm has input and reference points. The input points of KNN algorithm are the outputs of the previously determined formula, obtained when this formula is applied to features of segment under consideration. The reference points of KNN algorithm, on the other hand, are the outputs of the same formula, obtained when the formula is applied to the features of reference segments. For classifying a 26/24-channel segment, majority voting among the 26/24 decisions is employed to determine whether it is a spike or a nonspike segment.

## 5. Results and Discussion

We applied our spike detection approach on the data of 28 epileptic patients (mentioned in Section 3), of which eight were used in stage two and 20 in the classification stage. Two metrics were used to evaluate the performance, sensitivity, and specificity of the approach. Sensitivity represents the ratio of number of times the classifier makes correct positive decisions (i.e., detects spikes) to the total number of positive decisions. Specificity is the ratio of number of times the classifier makes correct negative decisions (i.e., detects spike-free segments) to the total number of negative decisions [38]. After data processing, eight patients were randomly selected to be used in the GP-based feature generation stage. Data of four of these patients were used as KNN reference points and the other four as unknown inputs with their ground truth values to find the best GP formula. The best formula was then applied to the new features of segments of the remaining 20-patient data to conduct the classification stage. Note that in the classification stage, we use the same reference points extracted from the data of four patients used to find the best GP formula.

Thereafter, the KNN is used to classify the 26/24-channel segment of data belonging to one of the eight regions of the brain using the new feature, where *k* = 100 nearest neighbor. The classifier results for each channel of a given segment are binary: “1” for channel with spikes and “0” for spike-free channel. A majority voting strategy is then used among the results of 26 channels to classify a segment of a particular region of the brain as spike or spike-free segment. The segment is labeled as a spike segment when the number of channels having spikes in the region under consideration is >13.

**Figure 5:**
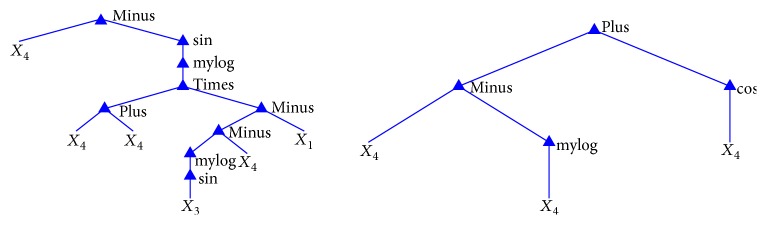
Examples of GP trees of the new feature.

The proposed algorithm achieved an average sensitivity and specificity of 91.75% and 92.99%, respectively, in 17 experiments.  Figure 5 presents two formulas' trees generated through GP evolution. In particular, these formulas are two out of 17 optimum formulas obtained by conducting 17 experiments. The right formula in Figure 5, which is using one feature *X*_4_ (standard deviation), gave the best performance out of the 17 formulas, as demonstrated (highlighted) in Table 2.

Khalid et al. [30] proposed a method for MEG spike detection using a common special pattern with linear discriminant analysis and achieved a sensitivity of 89.745% and a specificity of 89.154% on the same dataset. Applying the method of Ossadtchi et al. [31], which is a MEG spike detection algorithm using independent component analysis, on this dataset resulted in 83.278% sensitivity and 79.945% specificity. The results presented in this work demonstrate the potential of the proposed patient-independent method for use as a spike detection system.

## 6. Conclusion

In this work, we have presented a patient-independent spike detection approach based on statistical features and created the best possible features using GP. The approach consists of three stages: preprocessing, GP-based feature generation, and classification. GP with KNN is used to generate feature(s) that are more distinctive and reduce the input dimensions. The experimental results using real MEG data showed the effectiveness of the method in detecting interictal spikes with high sensitivity and specificity. The proposed spike detection method achieved an average sensitivity of 91.75% and an average specificity of 92.99%, respectively. This approach is a valuable tool for epileptologists, helping to accelerate the epilepsy diagnosis process.

## Figures and Tables

**Table 1:** GP parameters.

Parameter	Value
Population size	25
Number of generations	100
Maximum tree level	25
Crossover initial probability	90%
Mutation initial probability	10%
Functions	Plus, minus, times, sin, cos, log

**Table 2:** Results of 20 experiments.

Run	Sensitivity	Specificity
1	91.62%	93.63%
2	91.62%	93.90%
3	92.18%	91.64%
4	92.74%	92.71%
5	93.30%	92.41%
6	92.74%	91.70%
7	93.30%	91.40%
8	91.62%	93.42%
9	92.18%	92.73%
10	91.62%	93.34%
11	91.06%	93.54%
12	88.83%	94.17%
13	91.06%	93.54%
14	90.50%	93.80%
15	**93.85%**	**92.29%**
16	89.94%	93.73%
17	91.62%	92.92%
